# Vertical Distribution of Fruit Flies (Diptera: Drosophilidae) in Deciduous Forests in the Center of European Russia

**DOI:** 10.3390/insects14100822

**Published:** 2023-10-18

**Authors:** Nikolai G. Gornostaev, Alexander B. Ruchin, Mikhail N. Esin, Oleg E. Lazebny, Alex M. Kulikov

**Affiliations:** 1 N.K. Koltzov Institute of Developmental Biology RAS, 119334 Moscow, Russia; n_gornostaev@mail.ru (N.G.G.); amkulikov@gmail.com (A.M.K.); 2 Joint Directorate of the Mordovia State Nature Reserve and National Park “Smolny”, 430005 Saransk, Russia; ruchin.alexander@gmail.com (A.B.R.); esinmishka@gmail.com (M.N.E.)

**Keywords:** species diversity, stratification, fauna, seasonal dynamics, abundance, Republic of Mordovia

## Abstract

**Simple Summary:**

This study represents the first investigation of the vertical distribution of Drosophilidae in the European part of Russia. Traps suspended at various heights in deciduous forests were used to collect the specimens. Among the collected species, *Drosophila obscura* Fll. and *Scaptodrosophila rufifrons* Lw. were the most abundant. The highest total number of drosophilid flies (10,429 individuals) was captured at a height of 1.5 m, while the lowest number (5086 individuals) was recorded at 12 m. Five distinct vertical distribution patterns of drosophilids were identified throughout the season, demonstrating significant differences between mycetobiont and xylosaprobiont ecological groups. The maximum species diversity occurred in June and September.

**Abstract:**

Research of Diptera in temperate forests has demonstrated uneven vertical distributions of insects. In this study, we examined the vertical distribution, seasonal fluctuations, and species diversity of Drosophilidae species in the Mordovia State Reserve. This research marks the first exploration of drosophilid vertical stratification in the European part of Russia. Using traps, we collected flies in four deciduous forest sites between early June and mid-September in 2020. A total of 27,151 individuals from 10 genera and 34 drosophilid species were identified, with 6 species from 4 genera being new to the Republic of Mordovia. *Drosophila obscura* Fll. and *Scaptodrosophila rufifrons* Lw. were the most abundant species in traps. The total highest number of drosophilid flies (10,429 individuals) was captured at a height of 1.5 m, while the lowest number (5086 individuals) was recorded at 12 m. The average number of flies was 6240 and 5387 individuals at heights of 7.5 m and 3.5 m, respectively. However, the prevalence of drosophilid numbers at the 1.5-m height was not constant during the season. We found that in the second part of July the total fly counts at heights of 7.5 m and 12 m exceeded those at 1.5 m. We have described five different types of vertical distribution of drosophilids throughout the season, which differs markedly in mycetobionts and xylosaprobionts ecological groups. Species diversity demonstrated variations across different sites and tiers during the season, with peak diversity observed in June and September.

## 1. Introduction

In the center of the European part of Russia, the impact of anthropogenic load on forest ecosystems was very significant. However, insect communities in these ecosystems exhibit the capacity for recovery and persisting [1,2,3,4]. When studying insect communities in forests with a tropical climate, it turned out that many species display not only horizontal but also vertical distribution patterns. This stratification is due to the tiered composition of forest ecosystems [5,6]. Similar observations were made in temperate forests, where insects also exhibit distinct vertical distributions [7,8,9,10,11]. 

In recent years, the vertical distribution of various insects has been actively studied across different forest zones, ranging from tropical to temperate forests [12,13,14,15,16,17,18,19]. For example, the vertical stratification of beetles from the families Chrysomelidae, Cerambycidae, and Scarabaeidae has been actively studied in several countries [20,21,22]. In the temperate forests of Canada, the composition and dynamics of Coleoptera and Diptera communities varied significantly depending on the height of the traps [23].

The family Drosophilidae, widely known due to the huge role of its representatives in genetic research, includes more than 4600 described species worldwide [24,25,26]. This family of fruit flies is among the most ecologically diverse Diptera. Their larvae feed on various fruits and mushrooms, flowers, leaves, and plant tissues as miners, and in some cases are even predators [24,27,28,29,30,31,32].

Fruit flies are well-known forest inhabitants and, therefore, an interesting object for studying their stratification in forest biotopes. Studies have been conducted on the vertical distribution of drosophilids in Europe [33,34], North America [35], South America [36,37], Africa [17], Australia [38], and especially in Asia [39,40,41,42,43,44,45,46], but the European part of Russia remained unexplored.

The goal of our work was to study the vertical distribution of fruit flies, their species diversity, and seasonal changes in deciduous forests in the center of European Russia. Another goal was to carry out a comparison with the available data on the vertical distribution of drosophilids from the countries neighboring Russia.

## 2. Material and Methods

### 2.1. Study Area

The study was carried out in the Mordovia State Nature Reserve (European Russia), located in the southern boundary of the taiga zone (54°42′–54°56′ N, 43°04′–43°36′ E; up to 190 m a.s.l.). The Mordovia State Nature Reserve contains natural ecosystems in the center of the European part of Russia acknowledged as a hotspot for biodiversity [47,48,49,50]. The total area of the Protected Area is 321.62 km^2^ with forest communities covering 89.3% of this area. The reserve is located in a temperate zone. The coldest month, January, records average temperatures between −11.5 and −12.3 °C, while the warmest month, July, varies average temperatures of 18.9–19.8 °C. 

Insects were collected from the beginning of June to the middle of September in 2020. Field studies were conducted at 4 plots in the deciduous forests of the Republic of Mordovia (the center of European Russia). At each site within 20 m in a horizontal plane, 4 traps were installed on tree branches. To study vertical stratification, the traps were located at heights specific to deciduous forest tiers: 1.5, 3.5, 7, and 12 m above the ground. All experimental plots were located in the forest interior. The distance between the plots was at least 1.5 km from each other. The vegetation on each plot was to some extent different from other plots. At the plots, the first tier of the forest consisted of linden and oak with a projective coverage of 60%. The undergrowth layer was represented by maple, elm, buckthorn, rowan, small linden, and oak trees. The herbaceous tier was represented by various types of sedges (Carex), violets, lily of the valley, compound, and rosaceae plants [13]. 

### 2.2. Sampling

The sampling process involved the use of traps made from standard plastic 5-L water bottles, each of them with a cut-out window on one side, located 10 cm above the bottom [14,48]. These traps were hung singly on tree trunks at different heights: 1.5 m, 3.5 m, 7.5 m, and 12.0 m on neighboring trees growing at a distance of no more than 20 m from each other. To attract the flies, a mixture of beer, sugar, and honey was employed in the traps. Following collection, the samples were cleansed, immersed in alcohol, and transported to the laboratory. The fermentation period of the liquid bait was set to one day, and the sampling period varied from 6 to 15 days, depending on dry or wet weather; then, the bait was updated. All biotope-related research activities were conducted by A.B. Ruchin.

The identification of the collected flies was undertaken by N.G. Gornostaev, using a drosophilid key [51]. The systematic classification of Drosophilidae adhered to the interpretation provided by Grimaldi [52]. Species that were new to the region are indicated with an asterisk “*”. For statistical analysis, the data processing was supervised by A.M. Kulikov.

### 2.3. Statistical Analysis

Distribution diagrams detailing the number of drosophilid species across the four forest tiers and time intervals of observation were constructed using the Excel software. The species count data within collections were arranged by months, plots, and forest tiers.

The estimation of vertical species aggregation and the identification of horizontal stratification within the identified vertical distributions were accomplished through the application of Lloyd’s index of patchiness (LIP) and Kendall’s coefficient of concordance Wk, following the guidelines laid out by S. Tanabe [53]. Wk was also employed to assess the consistency of changes in species abundance across the four sites based on both forest tier and collection time. Calculations of coefficients, distribution diagrams of species values, and diversity indicators, such as Hill numbers and the Shannon index, were executed within Excel.

To validate the significance of factors, such as “site” and “height”, data were amalgamated based on the collection time and trap placement height, in the first instance, and on the collection time and site, in the second instance. This was achieved through nonparametric median and Kruskal–Wallis H tests.

The statistical significance of the influence exerted by all three factors and the interaction between the “season” and “height” factors on the fluctuations in species abundance was confirmed by subjecting the data to a multidimensional MANOVA (Multivariate Analysis of Variance). To determine the interdependency between the total species count in collections and all three factors, the analysis relied on the examination of contingency tables, with calculations involving Chi-Square test statistics and the coefficient of consistency. Parametric and nonparametric criterion calculations were carried out utilizing the IBM SPSS Statistics software (version 23, IBM Corp., Armonk, NY, USA).

## 3. Results

### 3.1. Faunistic Composition

Among the flies collected in traps in deciduous forests hanging at different heights, we found 5 genera and 9 species of subfamily Steganinae and 5 genera and 25 species of subfamily Drosophilinae. We found 6 new species in 4 genera in the Republic of Mordovia: *Stegana hypoleuca*, *Drosophila littoralis*, *D. subobscura*, *D. subsilvestris*, *Hirtodrosophila toyohiokadai*, and *Scaptomyza pallida* (Table 1).

### 3.2. Abundance and Seasonal Dynamics of Drosophilidae

As a result of the study in 2020, from the beginning of June to the middle of September, 27,151 individuals of drosophilids from 10 genera and 34 species were collected in 16 traps (Table 2).

Thus, 11 drosophilid species (*D. obscura*, *S. rufifrons*, *D. kuntzei*, *D. phalerata*, *D. testacea*, *D. histrio*, *P. semivirgo*, *L. quinquemaculata*, *D. subsilvestris*, *D. transversa* and *D. subobscura*) have been collected in the amount exceeding 100 individuals, so we designated them as the most common species in our materials. Among them, we consider as a truly mass species those whose total number in our collection exceeded 1000 individuals for each of them, namely *D. obscura* (11,311), *S. rufifrons* (5961), *D. kuntzei* (2313), *D. phalerata* (2067), *D. testacea* (1791), *D. histrio* (1100), and *P. semivirgo* (1070), as well as species with a moderate abundance of 100 to 1000 individuals for each of them, namely *L. quinquemaculata* (457), *D. subsilvestris* (323), *D. transversa* (255), and *D. subobscura* (187) (Table 2). The largest number of drosophilids was collected in early August and September and the smallest at the end of July (Table 2).

The remaining 23 species collected in the amount of less than 100 flies we consider as relatively rare (20–100 individuals) or extremely rare species (less than 20 individuals) in forest biotopes. In fact, this group of extremely rare species mainly consists of synanthropic species and species that are poorly attracted to these types of traps.

Interestingly, in our study the most common species of Drosophilidae demonstrate two different general patterns of seasonal dynamics, which well coincide with their ecological preferences. In the temperate zone, most drosophilid species belong to mycetobionts or xylosaprobionts, depending on the breeding sites of the larvae.

**Figure 1:**
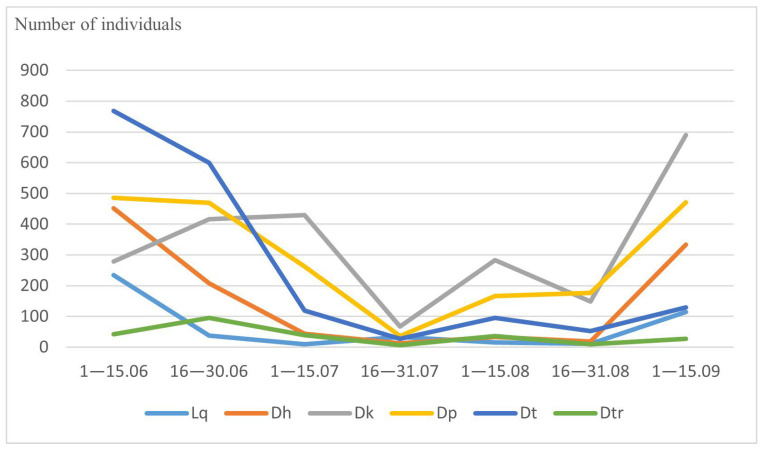
Seasonal dynamics of common mycetobiont drosophilid species. On the abscissa axis—the time of collection, on the ordinate axis—the total number of individuals. Lq—*L. quinquemaculata*, Dh—*D. histrio*, Dk—*D. kuntzei*, Dp—*D. phalerata*, Dt—*D. testacea*, Dtr—*D. transversa*.

**Figure 2:**
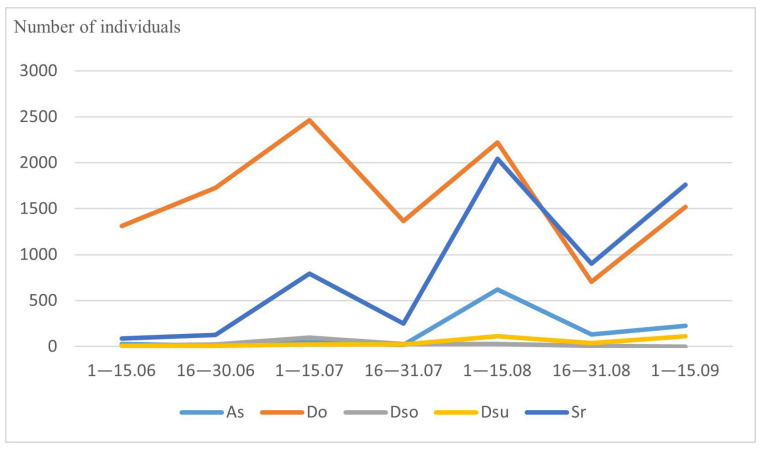
Seasonal dynamics of common xylosaprobiont drosophilid species. On the abscissa axis—the time of collection, on the ordinate axis—the total number of individuals. Ps—*P. semivirgo*, Do—*D. obscura*, Dso—*D. subobscura*, Dsu—*D. subsilvestris*, Sr—*S. rufifrons*.

Among our most common drosophilid species, *L. quinquemaculata*, *D. histrio*, *D. kuntzei*, *D. phalerata*, *D. testacea*, and *D. transversa* are typical mycetobionts breeding in various fungi. They showed the largest number in June, a sharp decrease at the end of July, and a noticeable increase in the number of individuals in September (Figure 1 and Table 2). On the contrary, drosophilid species of the xylosaprobiont group breeding in tree sap, rotting tissues under the bark, etc., namely *P. semivirgo*, *D. obscura*, *D. subobscura*, *D. subsilvestris*, and *S. rufifrons*, showed a low number of individuals in June and three peaks of abundancy at the beginning of July, August, and September (Figure 2 and Table 2).

### 3.3. Vertical Distribution of Drosophilidae

**Figure 3:**
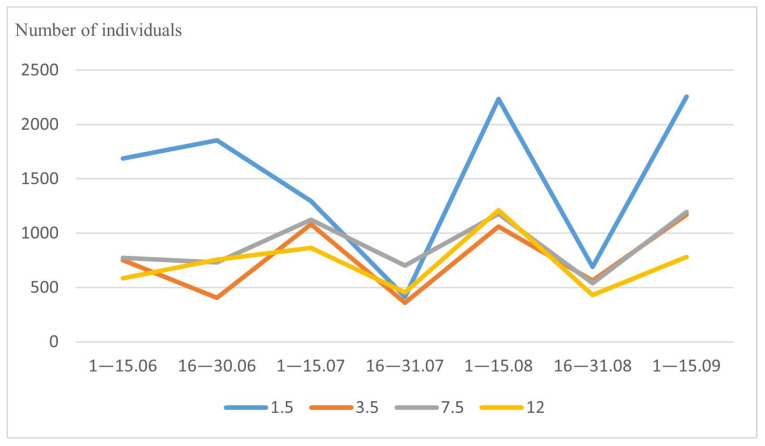
Seasonal changes in vertical distribution of Drosophilidae. On the abscissa axis—the time of collection, on the ordinate axis—the total number of individuals. Lines corresponding to a certain tier are marked with color: 1.5 m—blue, 3.5 m—red, 7.5 m—gray, 12 m—yellow.

In our study, the total highest number of drosophilid flies (10,429 individuals) was obtained at the height of 1.5 m, while the smallest number (5086 individuals) was found at the height of 12 m and the average numbers of the flies (6240 and 5387 individuals) at the heights of 7.5 m and 3.5 m, respectively. However, the prevalence of drosophilid abundance at the height of 1.5 m was not constant during the season. We found that in late July the abundance of these flies at the heights of 7.5 m and 12 m were noticeably higher than at the height of 1.5 m (703 and 455 vs. 412 individuals, respectively) (Figure 3). 

**Figure 4:**
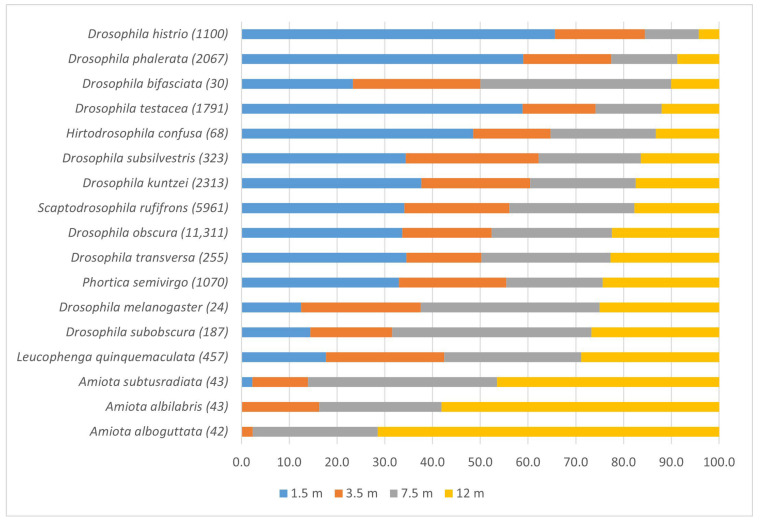
Distribution of drosophilid species in four tiers of the forest. The number on each tier is represented as a fraction of the total number of species-individuals in the collection. Species are ranked according to the increase in the proportion of numbers in the upper tier. The absolute number of the individuals is indicated in parentheses for each species. Species collected in an amount of less than 20 individuals are not represented.

In our research, most drosophilid species demonstrated an uneven distribution across the forest tiers (Figure 4). The increase in the relative abundance of the species on the upper tier (12 m) is accompanied, as a rule, by a decrease in its abundance on the lower tier (1.5 m). We found that *D. histrio*, *D. phalerata*, and *D. testacea* are the most common in the lower tier. The most uniform vertical distribution over the tiers in deciduous forests is demonstrated by *L. quinquemaculata* (Figure 4). 

The most obvious preference for the upper tier is demonstrated by *Amiota* species—*A. alboguttata*, *A. albilabris*, and *A. subtusradiata*. All of these species are xylosaprobionts that breed in tree sap and under the bark (Figure 4).

We also studied seasonal changes in the abundance of drosophilids depending on the tier for the common (more than 100 individuals collected) and relatively rare (20–100 individuals collected) species (Figure S1). A noticeable difference was found in the vertical distribution and preference of tiers during the season between the species of mycetobionts and xylosaprobionts; however, different patterns can also be distinguished within each of these ecological groups. Thus, we could describe five types of vertical distribution of Drosophilidae in temperate deciduous forests in the center of European Russia.

**Figure 5:**
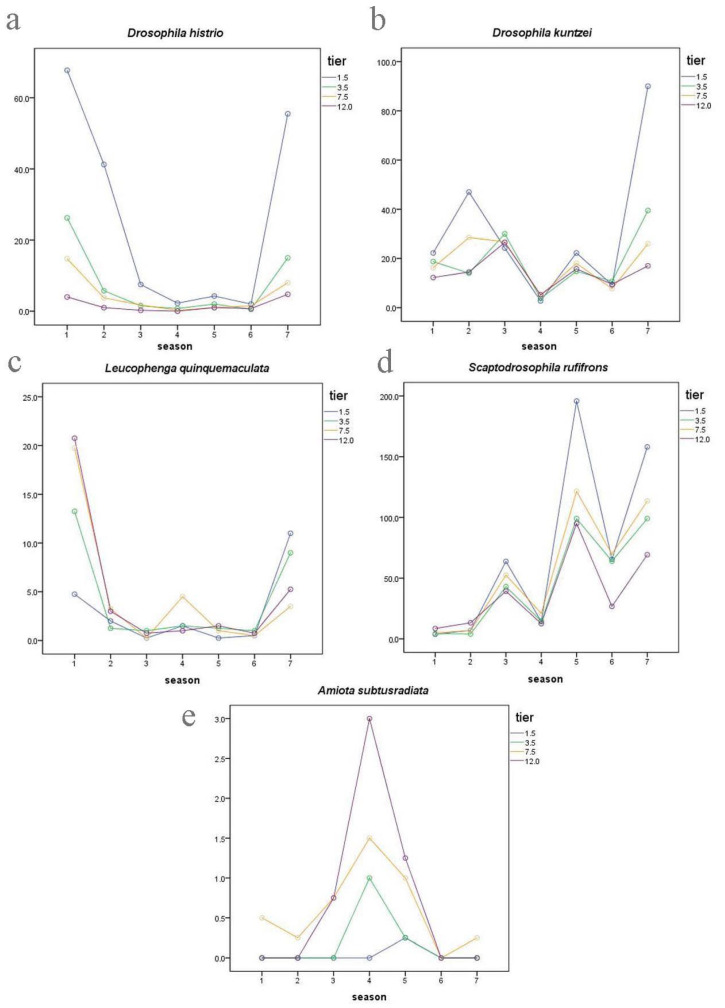
Vertical distribution and seasonal changes in the abundance of mycetobiont drosophilids (**a**–**c**) and xylosaprobiont drosophilids (**d**,**e**) depending on the tier: (**a**)—*D. histrio*, (**b**)—*D. kuntzei*, (**c**)—*L. quinquemaculata*, (**d**)—*S. rufifrons*, (**e**)—*A. subtusradiata*. There are seven collecting periods along the abscissa axis, from the first half of June to mid-September. On the ordinate axis are the marginal average numbers estimated by MANOVA. Lines corresponding to a certain tier are marked with color: 1.5 m—blue, 3.5 m—green, 7.5 m—yellow, 12 m—purple.

The first type of vertical drosophilid distribution is characterized by the fact that some species of the mycetobiont group, namely *D. histrio*, *D. phalerata*, and *D. testacea*, clearly prefer the lower tier of the forest throughout the season (Figure 5a and Figure S1). 

Here and further, only one of the most typical graphs of the dependence of the drosophilid abundance on the time of collection and the tier is presented for each group of species. A complete set of graphs for all species collected in the amount of more than 20 individuals is presented in the Supplementary Materials (Figure S1). 

However, two other common species from the ecological group of mycetobionts, *D. kuntzei* and *D. transversa*, showed a clear preference for the lower tier of the forest only in June and September, while in July and August, their vertical distribution across the tiers was approximately the same (Figure 5b and Figure S1). We assume that this is the second type of vertical distribution of drosophilids.

The third type of vertical distribution is represented by the mycetobiont species *L. quinquemaculata*, whose larvae live in various tinder fungi. This species showed the greatest abundance on the upper tiers of 7.5 m and 12 m in June, preferred the 7.5 m tier at the end of July, and the lower tiers of 1.5 m and 3.5 m in September (Figure 5c and Figure S1).

The next, fourth type of vertical distribution showed the species of drosophilids from the group of xylosaprobionts, *D. obscura*, *D. subsilvestris*, *P. semivirgo*, and *S. rufifrons* (Figure 5d and Figure S1). These species are characterized by a sharp increase in abundance in August, especially at the lower tier of 1.5 m, as well as a smaller peak in September. However, these species showed differences in abundance at the beginning of July—from the presence of a noticeable peak at the lower tier of 1.5 m in *D. obscura* and *S. rufifrons* to a small peak in *D. subsilvestris* and the absence of a peak in *P. semivirgo*.

Finally, the fifth type of vertical distribution of drosophilids is represented by species from the group of xylosaprobionts that prefer the highest tiers of the forest, *A. albilabris*, *A. alboguttata*, and *A. subtusradiata* (Figure 5e and Figure S1). For these species, the greatest abundance was observed in late July and early August.

Surprisingly, the vertical distribution of *D. subobscura*, a common species from the *D. obscura* group, is more similar to the fifth type noted for the *Amiota* species (Figure S1). These flies preferred the highest tiers of the forest, and the greatest abundance was observed at the height of 7.5 m in July.

The vertical distribution of a relatively rare species from the group of mycetobionts, *H. confusa*, seems to be close to the first type with the only difference in September, when the number of these flies on the upper tier slightly exceeded the number on the lower tier (Figure S1).

In the appendix, we have shown the vertical distribution of the other two relatively rare species, *D. bifasciata* and *D. melanogaster*, but it is difficult to analyze it due to the small number of individuals—30 and 24, respectively (Figure S1).

**Figure 6:**
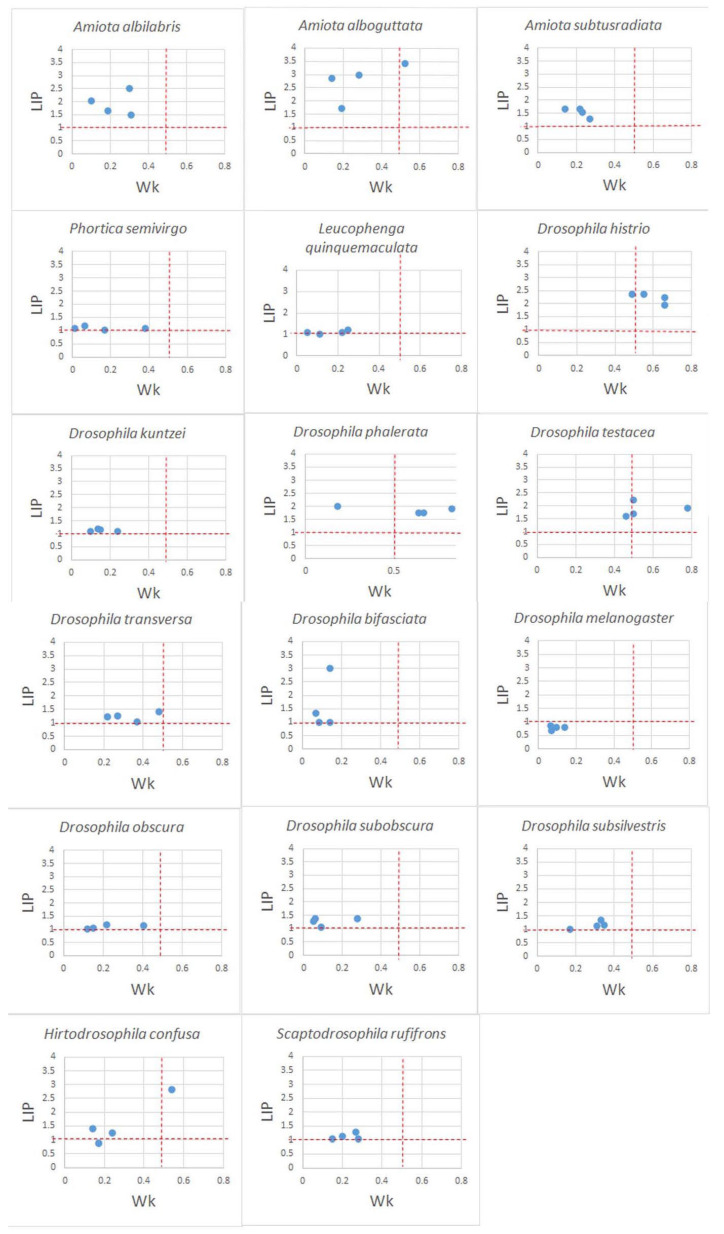
Vertical aggregation and horizontal stratification of drosophilids in the sites. The collection time at each site is used as repeats. The red dotted lines show critical indicators. Wk = 0.5 and LIP = 1, marking the transition from random values of aggregation and stratification to significant ones. The blue dots indicate the distribution of values of vertical aggregation and horizontal stratification of drosophilids in 4 sites.

The vertical aggregation of drosophilid populations, or the preference for habitat determined by the forest tier, was evaluated using the Lloyd’s index of patchiness (LIP). The degree of vertical aggregation was evaluated for each species, with the exception of a number of small ones. The estimates obtained on the basis of both the total summarized data for the entire collection period at all four sites (Table 3) and for each site separately (Figure 6) are given.

Almost all aggregation indices took values from one or more (up to 2.23 in *D. histrio* and 2.66 in *A. alboguttata*). These results suggest moderate to strong vertical aggregation of species, i.e., not a random preference of tiers. Significant concordance coefficients take values above 0.65 and indicate the similarity of the vertical distribution of drosophilids of the corresponding species in different sites according to the total data for the season. As a rule, significant estimates of Wk correspond to high indicators of LIP aggregation indices. *A. albilabris*, *A. alboguttata*, *A. subtusradiata*, *D. histrio*, *D. phalerata*, and *D. testacea* have high rates of stratification and aggregation. High stratification and intermediate aggregation rates are characteristic of *D. kuntzei*, *D. obscura*, and *S. rufifrons*.

This means that each of the species from the first group had a similar distribution of the total number for the entire period of collection on different tiers at each of the sites, and the distribution over different tiers differed sharply. The species from the second group also had a similar distribution by tiers at different sites, but their numbers on different tiers changed gradually, without sharp differences.

Below are the data for the species for each site when the collection time is used as repeats. The decrease in the values of the concordance coefficients in most cases is associated with a decrease in the sample for species whose total number was less than 400 specimens, or with sharp seasonal changes in the preferences of tiers (Figure 6).

In contrast to the estimates obtained for both on the basis of total summarized data for the entire collection period at all four sites (Table 2), the stratification values at individual sites were reduced in most species. High values of Wk were preserved in *D. histrio*, *D. phalerata*, and *D. testacea*. Since 15-day collection periods played the role of repeats in this analysis, seasonal changes in the numbers and changes in the tier preferences may be associated with such differences. Estimates of vertical aggregation of species remained at a level comparable to estimates based on aggregate data (Figure 6).

To check the effect of the “site” factor on the consistency of changes in the abundance of the species at different tiers, we analyzed the stratification values using the height of traps as repeats and sites as factors and summed up the number of drosophilid individuals for each trap for the entire collection period (Table S1).

At different sites, the number of species-individuals in most cases differed markedly, which was reflected in the fluctuations in the number on each tier. The fluctuations in the number on different tiers relative to the number on each site were similar, which explains the pronounced stratification according to the total summarized data. On the contrary, the fluctuations in the number at different sites relative to the total number on the tier at all sites were random. 

As a result, we received confirmation of random fluctuations in the abundance at each site, separately for each tier, which implies a low significance of the “site” factor. The exceptions are three species, *A. alboguttata*, *D. kuntzei*, and *D. phalerata*, for which significant concordance coefficients Wk were obtained. Obviously, for these species, the changes in abundance on the sites were synchronized with the changes in abundance on the tiers.

The consistency of changes in the total abundance of species in the tiers of plots depending on the time of the season was also checked, using seasons as repeats and sites as factors (Table S1). Seasonal changes in the abundance of most species at all four sites were coordinated, which indicates the importance of the “season” factor. The consistency of abundance changes at different sites depending on the season has not been confirmed for three species—*A. alboguttata*, *D. obscura*, and *D. subobscura*.

To confirm the significance of the influence of the factors “site” and “tier” on changes in the abundance of drosophila species over the entire observation period, nonparametric tests were used: median and Kruskel–Wallace (Table 4). The first test evaluates the significance of differences in medians in the compared samples and the second the general similarity of the distributions of the compared samples. A significant effect of the site on the abundance is shown only for *D. kuntzei*. This result confirms the earlier conclusion about the low significance of the “site” factor and the possibility of combining data on this factor. On the contrary, the influence of the “tier” factor on population dynamics turned out to be significant for half of the drosophilid species.

To check the role of all factors—site, tier, season, and the interaction of factors tier and season—a multidimensional analysis of the variance of rank-transformed data on the abundance of species was carried out, including all three factors and the specified interaction between them (Table 5 and Table 6).

The high significance of all elements of the linear model, the low values of the Wilkes Lambda, and the high values of the Hotelling Trace for the “season” effect and the effect of the interaction of the “season” and “tier” factors show an adequate assessment by the model of the variability of these factors. The Wilkes Lambda and Hotelling Trace estimates for the “tier” and “site” factors have weaker indicators. The Wilkes Lambda value shows the proportion of unexplained variance for this factor (independent variable), and the values indicate that for both factors, most of the variance is explained by the model. The estimates of the influence of factors on the abundance of species obtained in the model are given in Table 6.

The coefficient of determination shows the explained proportion of variance of dependent variables and abundance of species. The model adequately explains most of the variability in abundance of most species. A significant influence of the “site” factor was shown for three species, including *D. kuntzei*, *D. obscura*, and *D. subobscura*, for which the high significance of this factor and the violation of the coordinated change in abundance at different sites during the season were shown above. More than half of the species show the dependence of abundance on season and tier and almost all, with the exception of *A. alboguttata* and *D. melanogaster*, on the interaction of these factors.

### 3.4. Species Diversity of Drosophildae

**Figure 7:**
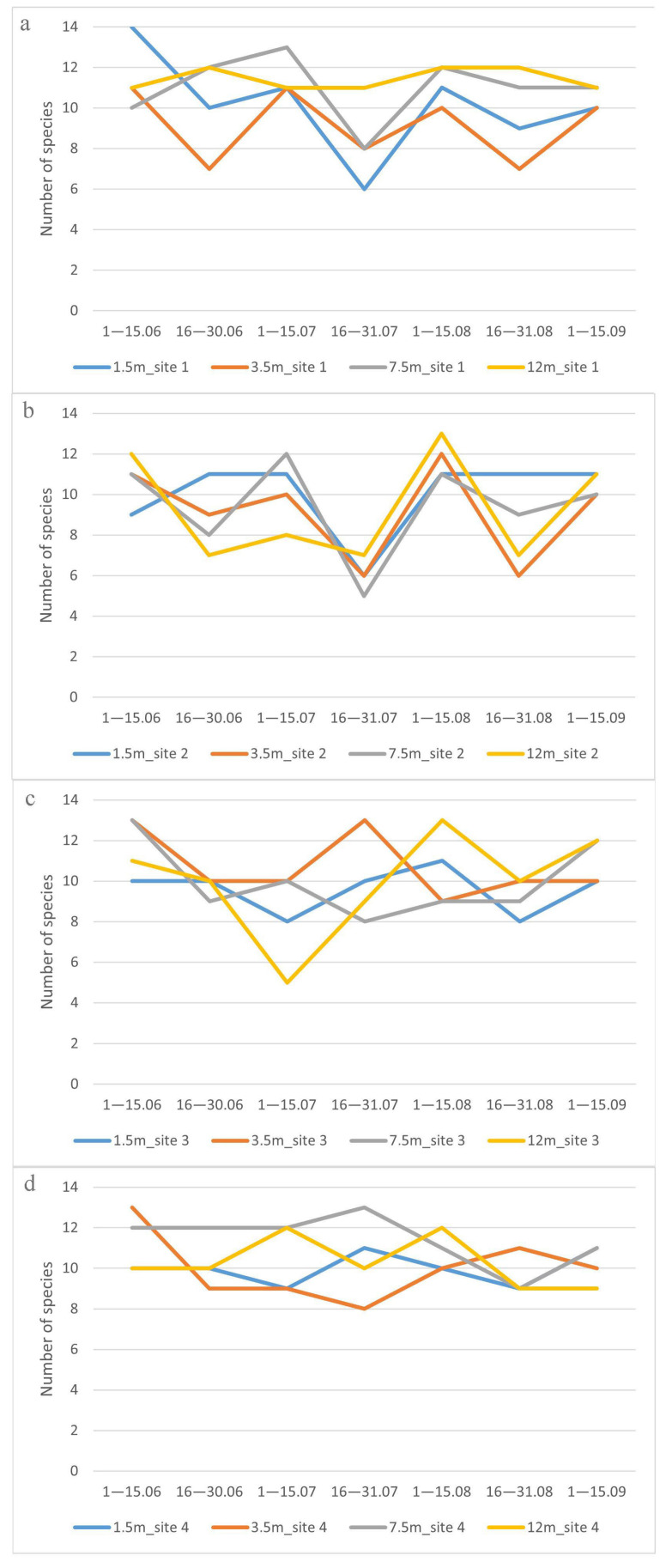
Seasonal dynamics of drosophilid species diversity: (**a**)—site 1, (**b**)—site 2, (**c**)—site 3, (**d**)—site 4. On the abscissa axis—the time of collection, on the ordinate axis—the number of species. The tiers are shown by color lines.

The diversity of the drosophilid species varied in different tiers of the forests from early June to mid-September (Figure 7). We also found a noticeable difference in species diversity between the four forest sites where the traps were hung.

Interestingly, as can be seen from Figure 7, the most coordinated seasonal changes in species diversity at all tiers of the forest occurred at sites 1 and 2, and at sites 3 and 4, these seasonal changes in species diversity had different directions at different tiers.

Thus, we come to the conclusion that when assessing seasonal changes in the species diversity of drosophilids, the “site” is of great importance, in contrast to the low significance of the “site” when analyzing the dependence of drosophilid abundance on different factors, as shown earlier (Table 4).

When assessing the diversity of species, combined data on four forest sites were used. Hill numbers represent the weighted average number of species, in our case distributed depending on the tier and the time of collection. At q = 1, the average geometric value is estimated, and each species is weighted in proportion to its number. At q = 2, the arithmetic mean is estimated, and the weight given to rare species is reduced. The results obtained show a relatively low proportion (approximately 25% in accordance with the difference in numbers at q = 1 and q = 2) of rare species in the studied biotope. The maximum species diversity is observed in early June and in September.

**Figure 8:**
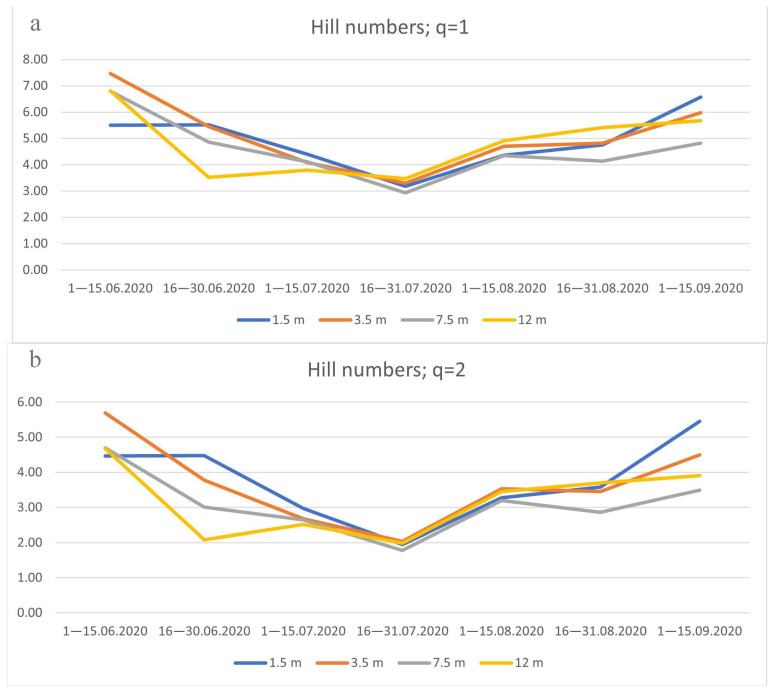
The average number of species, in the order of diversity, q = 1 (**a**) and q = 2 (**b**), during seasonal changes in the number of species on the four tiers. On the abscissa axis—the time of collection, on the ordinate axis—the estimated number of species. The colored lines show the tiers of trap locations.

**Figure 9:**
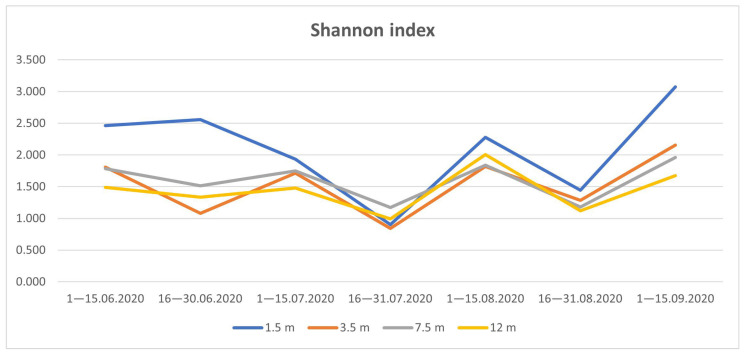
The change in the Shannon index when assessing the diversity of drosophilid species depending on the season and tier.

The exponent ^1^D (Hill numbers at q = 1) is exponentially dependent on the Shannon index. As shown by the Shannon index, the greatest species diversity of Drosophilidae is observed in early summer and autumn, while by the end of July and the end of August, there is a noticeable decrease in diversity, proportional at all tiers (Figure 8 and Figure 9).

When taking into account all species, regardless of their abundance in the collection, the influence of the season on the total number of species in the collection is shown (Table 7). When removing species with the number of individuals less than 20 specimens, dependence on both site and tier is manifested. All factors exhibit moderate conjugacy with the indicators of the number of species in the collection.

## 4. Discussion

The fauna of Drosophilidae of the Republic of Mordovia is under active research in the last few years. Initially, the insect fauna was studied in the post-fire forest recovery process and included 15 species in 6 genera of Drosophildae [48]. The next study focused on the investigation of seasonal dynamics of drosophilids in five different types of forests, and the faunistic list was increased to 30 species in 9 genera [54]. The complete faunistic list of Drosophilidae of the Republic of Mordovia currently consists of 36 species in 10 genera, with *Stegana coleoptrata* (Scopoli, 1763) and *Scaptomyza unipunctum* (Zetterstedt, 1847) recorded earlier [54].

In our study, we collected 27,151 individuals belonging to 10 genera and 34 species of Drosophilidae at four heights by traps in four sites of deciduous forests from the beginning of June to the middle of September. This is the first study of the vertical distribution of fruit flies in the European part of Russia.

We have identified five types of vertical distribution of drosophilids among the most common (11 species) and 4 relatively rare species (*A. albilabris*, *A. alboguttata*, *A. subtusradiata*, and *H. confusa*). It turned out that the majority of drosophilid species (10 species) in the central part of European Russia preferred the lower tier of the forest, and 5 species preferred the crown of trees at least in some periods.

Three *Amiota* species, *A. albilabris*, *A. alboguttata*, and *A. subtusradiata*, preferred the upper tier throughout the season, this preference for a tree crown has already been noted for many species of this genus in Japan [39] and for *A. alboguttata* in Scotland [55]. The canopy drosophilid species in boreal and cool-temperate forests are mostly sap and decayed bark feeders [56], it means they belong to xylosaprobiont group. Another species of this group, *D. subobscura*, also preferred the upper tiers from early June to the end of August when we were able to collect it. Finally, one species of mycetobiont group, *L. quinquemaculata*, demonstrated a preference for the upper tiers only in June. The larvae of this species breed in various tinder fungi, and apparently, these flies are aggregated mostly in the upper tiers in early summer when the young tinder appear on the trunks. 

The largest drosophilid group that preferred the lower tier consists of six mycetobiont species and four xylosaprobiont species. Larvae of mycetobiont species *D. histrio*, *D. kuntzei*, *D. phalerata*, *D. testacea*, *D. transversa*, and *H. confusa* breed mainly in various basidiomycetes, although adult flies have also been observed on some tinder fungi. Larvae of xylosaprobiont species, *P. semivirgo*, *D. obscura*, *D. subsilvestris*, and *S. rufifrons*, feed and breed in fermenting tree sap and decaying tissues under the bark.

For Drosophilidae in England, it has been suggested that the ecological division into different tiers between related species may be an important factor due to reduced competition for food and breeding sites. In this work, the difference between similar species is noted: *D. obscura* clearly preferred the crown, and the abundance of *D. subobscura* was approximately the same between the upper and lower tiers of the forest [33]. 

In our study, however, we observed the opposite pattern. *D. subobscura* clearly preferred the upper tiers, and *D. obscura* preferred the lower tier or was distributed between tiers in equal numbers. This example confirms the partial ecological division into different tiers in closely related species, *D. obscura* and *D. subobscura*; although, the direction of this division is opposite in England and the central part of European Russia.

To date, only one study has examined the vertical distribution of Drosophilidae in the Asian part of Russia [57]. The authors compared the vertical distribution of drosophilids in the northern birch forest (Yakutia) and the temperate birch forest (Hokkaido, Japan). Unfortunately, it is difficult to compare our results with this study, since the species composition of the fruit flies of Yakutia turned out to be much smaller (13 species) and very different from the fauna of Mordovia. For example, the two most abundant species in Yakutia are *D. bifasciata* and *D. funebris*, which are present in our collections in the amount of 30 and 1 individuals, respectively. The only species with a similar vertical distribution and similar abundance in collections (245 individuals in Republic of Yakutia and 255 individuals in Republic of Mordovia) was *D. transversa*, which in both regions preferred the lower tiers of the forest. However, some similarity was also found between our results and the vertical distribution of fruit flies in the temperate forest of Japan. In both cases, *Amiota* species preferred the upper tiers, while *D. histrio* was the most abundant at the height of 1.5 m. 

In addition, in Japan, the autumn invasion of some mycetobiont species from the lower to the upper tiers was noted, presumably in connection with the preparation for diapause [39]. However, we have not observed such an invasion of mycetobiont drosophilids, with the exception of *H. confusa*.

Among the 34 species of drosophilids collected in the Republic of Mordovia, 5 species of the genus *Drosophila* (*D. busckii*, *D. funebris*, *D. hydei*, *D. immigrans*, *D. melanogaster*) are synanthropic and can be found in people’s homes, outbuildings, food markets, etc. They live and breed in places where they can find fermenting and rotting fruits and vegetables, wine, beer, and juices [58,59,60,61]. 

In our collection, the synanthropic species *D. busckii*, *D. funebris*, *D. hydei*, and *D. immigrans* were found in very small numbers (1–4 individuals). Another synanthropic species, *D. melanogaster*, was collected in an amount of 24 individuals, which was also insufficient to obtain a clear diagram of its vertical distribution during the season. We consider these findings as accidental invasions of synanthropic species from human habitats into the forest, probably as a result of migration or wind transport.

In our work, we studied the vertical distribution of drosophilids collected only from the beginning of June to the middle of September for one year. In the future, it would be interesting to study the material collected also in May and October, when fruit flies may still be active in our climate, as well as to make a comparison of the vertical distribution in different years.

In recent years, studies have been conducted on the seasonal dynamics and species diversity of drosophilids in semi-natural biotopes—in the vineyards of France [62] and in the vineyards and fruit orchards of Turkey [63,64]. It can be concluded that the species diversity of drosophilids collected by traps is significantly higher in the natural biotopes of the center of the European part of Russia (34 species) compared with semi-natural biotopes even in countries with warmer climates—France (17 species) and Turkey (11 and 13 species, respectively). This fact underlines the importance of protected areas, such as the Mordovia State Nature Reserve, in conservation of the species diversity of various animals and plants and natural ecosystems in general.

## 5. Conclusions

A total of 27,151 individuals belonging to 10 genera and 34 species of drosophilid flies were identified, *Drosophila obscura* and *Scaptodrosophila rufifrons* were the most abundant species in traps. The total highest number of drosophilid flies (10,429 individuals) was obtained at the height of 1.5 m, while the smallest number (5086 individuals) was found at the height of 12 m. However, the prevalence of the drosophilid number at the height of 1.5 m was not constant during the season. We found that in the second part of July the total amounts of these flies at the heights of 7.5 m and 12 m were noticeably higher than at the height of 1.5 m. We have described five different types of vertical distribution of drosophilids throughout the season, which differs markedly in the ecological groups of mycetobionts and xylosaprobionts. We have identified five types of vertical distribution of drosophilids and found that the majority of common drosophilid species (10 species) in the central part of European Russia preferred the lower tier of the forest, and 5 species preferred the crown of trees at least in some periods. A significant influence of the “site” factor was shown only for three species, more than half of the species showed the dependence of abundance on season and tier, and almost all drosophilid species showed it on the interaction of these factors. Species diversity varied between different sites and tiers during the season. The maximum species diversity is observed in early June and in September, while at the end of July and at the end of August there is a noticeable decrease in diversity. When assessing seasonal changes in the species diversity of drosophilids, the “site” is of great importance, in contrast to the low significance of the “site” when analyzing the dependence of drosophilid abundance on different factors.

## Figures and Tables

**Table 1:** The faunistic list of drosophilid flies in our collection.

Subfamily Steganinae	Subfamily Drosophilinae
1. *Amiota (Amiota) albilabris* (Roth in Zetterstedt, 1860)	1. *Chymomyza amoena* (Loew, 1862)
2. *Amiota (Amiota) alboguttata* (Wahlberg, 1839)	2. *Chymomyza caudatula* (Oldenberg, 1914)
3. *Amiota (Amiota) rufescens* (Oldenberg, 1914)	3. *Chymomyza costata* (Zetterstedt, 1838)
4. *Amiota (Amiota) subtusradiata* (Duda, 1934)	4. *Chymomyza fuscimana* (Zetterstedt, 1838)
5. *Phortica (Phortica) semivirgo* (Maca, 1977)	5. *Drosophila (Dorsilopha) busckii* (Coquillett, 1901)
6. *Gitona distigma* (Meigen, 1830)	6. *Drosophila (Drosophila) funebris* (Fabricius, 1787)
7. *Leucophenga maculata* (Dufour, 1839)	7. *Drosophila (Drosophila) histrio* (Meigen, 1830)
8. *Leucophenga quinquemaculata* (Strobl, 1893)	8. *Drosophila (Drosophila) hydei* (Sturtevant, 1921)
9. * *Stegana (Steganina) hypoleuca* (Meigen, 1830)	9. *Drosophila (Drosophila) immigrans* (Sturtevant, 1921)
	10. *Drosophila (Drosophila) kuntzei* (Duda, 1924)
	11. * *Drosophila (Drosophila) littoralis* (Meigen, 1830)
	12. *Drosophila (Drosophila) phalerata* (Meigen, 1830)
	13. *Drosophila (Drosophila) testacea* (von Roser, 1840)
	14. *Drosophila (Drosophila) transversa* (Fallen, 1823)
	15. *Drosophila (Sophophora) bifasciata* (Pomini, 1940)
	16. *Drosophila (Sophophora) melanogaster* (Meigen, 1830)
	17. *Drosophila (Sophophora) obscura* (Fallen, 1823)
	18. * *Drosophila (Sophophora) subobscura* (Collin in Gordon, 1936)
	19. * *Drosophila (Sophophora) subsilvestris* (Hardy et Kaneshiro, 1968)
	20. *Drosophila (Sophophora) tristis* (Fallen, 1823)
	21. *Hirtodrosophila confusa* (Staeger, 1844)
	22. * *Hirtodrosophila toyohiokadai* (Sidorenko,1990)
	23. *Hirtodrosophila trivittata* (Strobl, 1893)
	24. *Scaptodrosophila rufifrons* (Loew, 1873)
	25. * *Scaptomyza (Parascaptomyza) pallida* (Zetterstedt, 1847)

*—new species for the fauna of the Republic of Mordovia.

**Table 2:** Total number of drosophilid individuals collected in traps.

Species	1–15 June	16–30 June	1–15 July	16–30 July	1–15 August	16–30 August	1–15 September	Total Amount
*Amiota albilabris*	11	4	2	12	12	0	2	43
*Amiota alboguttata*	5	0	2	18	6	3	8	42
*Amiota rufescens*	0	0	0	0	3	0	0	3
*Phortica semivirgo*	25	6	48	18	618	132	223	1070
*Amiota subtusradiata*	2	1	6	22	11	0	1	43
*Gitona distigma*	7	0	0	0	0	2	3	12
*Leucophenga maculata*	1	0	0	0	0	0	2	3
*Leucophenga quinquemaculata*	234	38	9	34	16	11	115	457
*Stegana hypoleuca*	1	0	0	0	0	0	0	1
*Chymomyza amoena*	1	0	1	0	1	0	0	3
*Chymomyza caudatula*	13	1	0	0	0	0	0	14
*Chymomyza costata*	0	1	0	0	0	1	0	2
*Chymomyza fuscimana*	2	0	0	0	0	0	1	3
*Drosophila bifasciata*	6	3	19	2	0	0	0	30
*Drosophila busckii*	0	0	0	0	0	0	2	2
*Drosophila funebris*	1	0	0	0	0	0	0	1
*Drosophila histrio*	451	207	44	13	33	19	333	1100
*Drosophila hydei*	0	1	0	1	0	0	0	2
*Drosophila immigrans*	0	2	0	0	0	0	2	4
*Drosophila kuntzei*	278	416	430	67	283	149	690	2313
*Drosophila littoralis*	1	2	0	0	0	0	0	3
*Drosophila melanogaster*	7	5	3	0	4	4	1	24
*Drosophila obscura*	1313	1724	2464	1368	2219	705	1518	11,311
*Drosophila phalerata*	485	469	262	37	166	177	471	2067
*Drosophila subobscura*	8	22	97	29	25	6	0	187
*Drosophila subsilvestris*	9	10	21	22	114	36	111	323
*Drosophila testacea*	768	600	119	28	95	52	129	1791
*Drosophila transversa*	42	95	39	6	36	10	27	255
*Drosophila tristis*	0	0	2	0	0	0	0	2
*Hirtodrosophila confusa*	31	15	3	2	6	5	6	68
*Hirtodrosophila toyohiokadai*	0	1	0	0	0	1	0	2
*Hirtodrosophila trivittata*	4	0	1	0	0	0	2	7
*Scaptodrosophila rufifrons*	86	124	794	249	2045	904	1759	5961
*Scaptomyza pallida*	2	0	0	0	0	0	0	2
Number of species	27	22	20	17	18	17	21	34
Total number of individuals	3794	3747	4366	1928	5693	2217	5406	27,151

**Table 3:** Vertical aggregation and horizontal stratification of drosophilids.

Species	Stratification in Each Site *				LIP
	Wk	χ^2^	d.f.	*p*	
*Amiota albilabris*	**0.92**	**1.87**	3	**0.0021**	1.87
*Amiota alboguttata*	**0.91**	**2.66**	3	**0.0023**	2.66
*Amiota subtusradiata*	**0.89**	**1.64**	3	**0.0026**	1.64
*Phortica semivirgo*	0.29	1.05	3	0.3260	1.05
*Leucophenga quinquemaculata*	0.40	1.03	3	0.1870	1.03
*Drosophila histrio*	**0.93**	**2.23**	3	**0.0112**	2.23
*Drosophila kuntzei*	**0.85**	**1.12**	3	**0.0167**	1.12
*Drosophila phalerata*	**0.75**	**1.85**	3	**0.0293**	1.85
*Drosophila testacea*	**0.70**	**1.82**	3	**0.0384**	1.82
*Drosophila transversa*	0.23	1.08	3	0.4402	1.08
*Drosophila bifasciata*	0.28	1.11	3	0.3394	1.11
*Drosophila melanogaster*	0.34	1.00	3	0.2561	1.00
*Drosophila obscura*	**0.68**	1.06	3	**0.0440**	1.06
*Drosophila subobscura*	0.41	1.22	3	0.1755	1.22
*Drosophila subsilvestris*	0.43	1.09	3	0.1646	1.09
*Hirtodrosophila confusa*	0.58	1.36	3	0.0735	1.36
*Scaptodrosophila rufifrons*	**0.73**	1.08	3	**0.0336**	1.08

Significant concordance coefficients Wk are highlighted in bold. Stratification in each site *—trap heights are factors, sites for the set of traps are repeats. Species whose number is less than 20 individuals for the entire season are not included.

**Table 4:** Significance of the factors “site” and “tier” according to the total data.

Species	Site *		Tier *	
	Median Test *p*	K–W H Test *p*	Median Test *p*	K–W H Test *p*
*Amiota albilabris*	0.572	0.613	**0.046**	**0.014**
*Amiota alboguttata*	0.572	0.503	**0.019**	**0.015**
*Amiota subtusradiata*	0.859	0.673	**0.005**	**0.016**
*Phortica semivirgo*	0.425	0.420	**0.031**	0.193
*Leucophenga quinquemaculata*	0.185	0.095	0.077	0.287
*Drosophila histrio*	0.572	0.907	**0.019**	**0.004**
*Drosophila kuntzei*	**0.019**	**0.015**	0.572	0.282
*Drosophila phalerata*	0.425	0.617	**0.031**	**0.011**
*Drosophila testacea*	0.261	0.533	0.112	**0.022**
*Drosophila transversa*	0.261	0.205	0.572	0.405
*Drosophila bifasciata*	0.362	0.273	0.785	0.373
*Drosophila melanogaster*	0.572	0.304	0.572	0.339
*Drosophila obscura*	0.572	0.522	**0.019**	0.051
*Drosophila subobscura*	0.859	0.639	0.077	0.124
*Drosophila subsilvestris*	0.572	0.417	0.572	0.184
*Hirtodrosophila confusa*	0.362	0.555	**0.022**	**0.037**
*Scaptodrosophila rufifrons*	0.572	0.687	**0.019**	**0.023**

The values of the p-criterion less than 0.05 are highlighted in bold. Site *—samples are combined by seasons and tiers; Tier *—samples are combined by sites and seasons. K-W H test—Kruskal–Wallis H test.

**Table 5:** Multidimensional criteria of the applied linear model.

Effect	Criteria	Value	F	df1	df2	Significance
Free member	Wilkes’ Lambda	0.072	51,844	17.000	68.000	0.000
	Hotelling Trace	12.961	51,844	17.000	68.000	0.000
Season	Wilkes’ Lambda	0.000	3.124	357.000	956.097	0.000
	Hotelling Trace	31.599	5.852	357.000	1124.000	0.000
Tier	Wilkes’ Lambda	0.204	2.817	51.000	203.253	0.000
	Hotelling Trace	2.455	3.210	51.000	200.000	0.000
Site	Wilkes’ Lambda	0.294	2.026	51.000	203.253	0.000
	Hotelling Trace	1.628	2.128	51.000	200.000	0.000
Season * tier	Wilkes’ Lambda	0.000	3.347	476.000	1044.741	0.000
	Hotelling Trace	34.216	4.681	476.000	1107.000	0.000

F—F statistic; df—degrees of freedom; df1—df of the hypothesis; df2—error df from the F-approximation to the distribution of MANOVA algorithms; *—a combination of the factors season and tier.

**Table 6:** Significance of the influence of factors and a combination of the factors season * tier on the abundance of Drosophilidae.

	Adjusted Model				Season			Tier			Site			Season * Tier		
Species	R^2^ Corrected	d.f.	F	*p*	d.f.	F	*p*	d.f.	F	*p*	d.f.	F	*p*	d.f.	F	*p*
*Phortica semivirgo*	0.817	29	18.279	**0.000**	21	13.401	**0.000**	3	1.698	0.174	3	2.058	0.155	28	12.685	**0.000**
*Leucophenga quinquemaculata*	0.757	29	13.015	**0.000**	21	8.755	**0.000**	3	3.437	**0.021**	3	0.209	0.649	28	8.728	**0.000**
*Drosophila histrio*	0.746	29	12.341	**0.000**	21	4.539	**0.000**	3	10.432	**0.000**	3	0.380	0.539	28	9.580	**0.000**
*Drosophila kuntzei*	0.785	29	15.086	**0.000**	21	10.591	**0.000**	3	1.827	0.149	3	18.486	**0.000**	28	6.723	**0.000**
*Drosophila subobscura*	0.65	29	8.170	**0.000**	21	3.021	**0.000**	3	3.345	**0.023**	3	7.374	**0.008**	28	5.908	**0.000**
*Drosophila phalerata*	0.765	29	13.575	**0.000**	21	2.588	**0.001**	3	14.893	**0.000**	3	4.167	**0.044**	28	8.183	**0.000**
*Drosophila testacea*	0.787	29	15.280	**0.000**	21	3.056	**0.000**	3	11.045	**0.000**	3	3.315	0.072	28	11.636	**0.000**
*Drosophila transversa*	0.484	29	4.627	**0.000**	21	1.947	**0.017**	3	1.647	0.185	3	1.151	0.286	28	2.320	**0.002**
*Drosophila obscura*	0.758	29	13.067	**0.000**	21	1.814	**0.030**	3	4.157	**0.009**	3	6.414	**0.013**	28	2.641	**0.000**
*Drosophila subsilvestris*	0.679	29	9.172	**0.000**	21	6.430	**0.000**	3	1.730	0.167	3	0.449	0.505	28	4.995	**0.000**
*Scaptodrosophila rufifrons*	0.762	29	13.341	**0.000**	21	9.793	**0.000**	3	2.230	0.091	3	0.547	0.462	28	6.530	**0.000**
*Amiota albilabris*	0.48	29	4.571	**0.000**	21	1.137	0.329	3	7.460	**0.000**	3	1.840	0.179	28	3.469	**0.000**
*Amiota alboguttata*	0.158	29	1.722	**0.029**	21	1.322	0.185	3	3.714	**0.015**	3	0.985	0.324	28	1.379	0.133
*Amiota subtusradiata*	0.411	29	3.699	**0.000**	21	1.672	0.052	3	4.472	**0.006**	3	0.347	0.557	28	2.870	**0.000**
*Drosophila bifasciata*	0.274	29	2.457	**0.001**	21	1.058	0.408	3	0.698	0.556	3	1.203	0.276	28	2.096	**0.005**
*Drosophila melanogaster*	0.157	29	1.721	**0.029**	21	0.589	0.916	3	1.303	0.279	3	0.074	0.787	28	1.019	0.456
*Hirtodrosophila confusa*	0.532	29	5.398	**0.000**	21	3.884	**0.000**	3	7.858	**0.000**	3	1.712	0.194	28	3.447	**0.000**

R^2^—coefficient of determination, d.f.—degrees of freedom, F—F-criterion. The values of *p* < 0.05 are marked in bold. *—a combination of the factors season and tier.

**Table 7:** Dependence of the species diversity of drosophilids on the site, tier, and season.

Factor	Criteria	N			N *		
			d.f.	*p*		d.f.	*p*
site	χ^2^	33.799	30	0.289	**38.952**	**27**	**0.043**
	c	0.481		0.280	0.508		0.064
tier	χ^2^	27.975	30	0.572	**38.075**	**27**	**0.050**
	c	0.447		0.598	0.504		0.054
season	χ^2^	**79.034**	**60**	**0.037**	**75.823**	**54**	**0.027**
	c	0.643		0.037	0.635		0.017

N *—species with the number of individuals less than 20 specimens are excluded from the analysis. Significant values of factors are given in bold.

## Data Availability

The data presented in the study are available in the article.

## Supplementary Materials

The following supporting information can be downloaded at: https://www.mdpi.com/article/10.3390/insects14100822/s1, Table S1: Consistency of changes in the abundance of each species in the sites depending on the tier and depending on the time of collection of drosophilids, Figure S1: Seasonal changes in the abundance of drosophilids depending on the tier. There are seven collecting periods along the abscissa axis, from the first half of June to mid-September.
