# Supplementary material for: Vertical Distribution of Fruit Flies (Diptera: Drosophilidae) in Deciduous Forests in the Center of European Russia
Source: Insects. 2023 Oct 18;14(10):822. doi: 10.3390/insects14100822 (PMC10607236; doi:10.3390/insects14100822)
Supplement: Supplementary file 1 [file insects-14-00822-s001.zip › insects-2609723-supplementary.pdf]

Supplementary materials

Table S1. Consistency of changes in the abundance of each species in the sites depending on the tier and depending on the time of collection of drosophilids.

Species	stratification of each level*				stratification of the abundance of samples at the sites by the time of collection *			
	W	χ2	d.f.	p	W	χ2	d.f.	p
Amiota albilabris	0.41	4.86	3	0.1823	**0.55**	13.09	6	**0.0416**
Amiota alboguttata	0.83	13.21	3	**0.0042**	0.21	4.98	6	0.5464
Amiota subtusradiata	0.32	3.82	3	0.2816	**0.60**	14.29	6	**0.0266**
Amiota semivirgo	0.20	2.38	3	0.4974	**0.89**	21.32	6	**0.0016**
Leucophenga quinquemaculata	0.33	3.92	3	0.2702	**0.83**	19.89	6	**0.0029**
Drosophila histrio	0.48	5.70	3	0.1272	**0.89**	17.86	6	**0.0066**
Drosophila kuntzei	1.00	12.00	3	**0.0074**	**0.88**	21.23	6	**0.0017**
Drosophila phalerata	0.68	8.10	3	**0.0440**	**0.82**	19.71	6	**0.0031**
Drosophila testacea	0.56	6.69	3	0.0825	**0.78**	18.75	6	**0.0046**
Drosophila transversa	0.48	5.70	3	0.1272	**0.88**	21.21	6	**0.0017**
Drosophila bifasciata	0.34	4.09	3	0.2519	**0.62**	14.83	6	**0.0216**
Drosophila melanogaster	0.24	2.83	3	0.4186	**0.62**	14.93	6	**0.0208**
Drosophila obscura	0.33	3.90	3	0.2725	0.37	8.90	6	0.1793
Drosophila subobscura	0.24	2.85	3	0.4153	0.47	11.36	6	0.0779
Drosophila subsilvestris	0.30	3.60	3	0.3080	**0.86**	17.19	6	**0.0086**
Hirtodrosophila confusa	0.16	1.92	3	0.5892	**0.85**	20.48	6	**0.0023**
Scaptodrosophila rufifrons	0.15	1.80	3	0.6149	**0.66**	15.78	6	**0.0150**

Stratification in each level* - sites are factors, trap heights are repeats; stratification of the abundance of samples at the sites by the time of collection * - sites are factors, collection seasons are repeats. Significant indicators of Wk are highlighted in bold.

Amiota albilabris

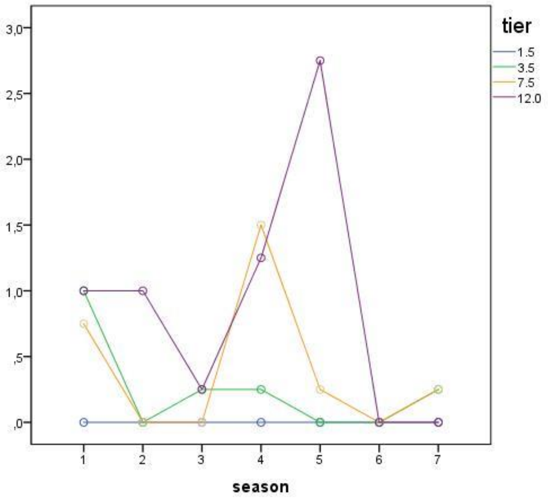

Amiota alboguttata

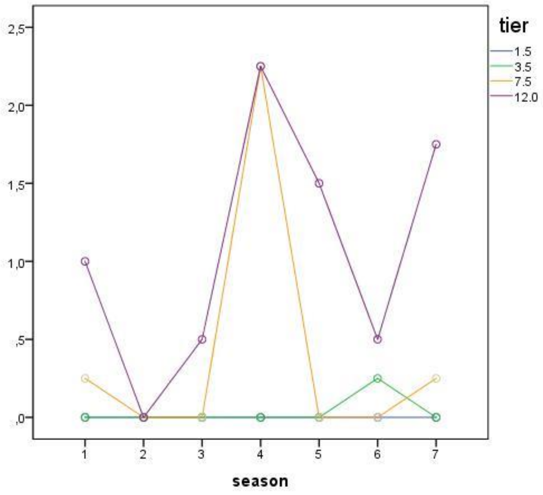

Amiota subtusradiata

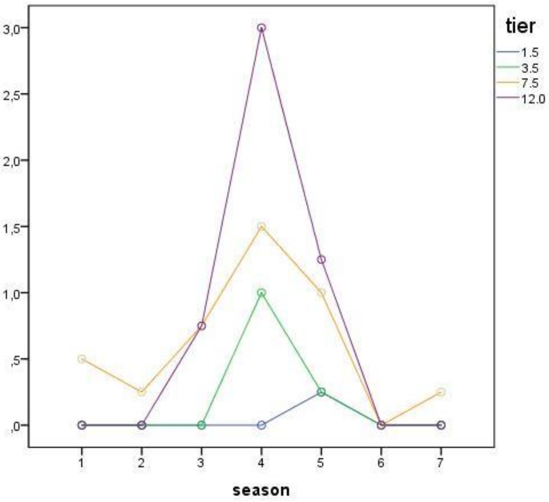

Drosophila bifasciata

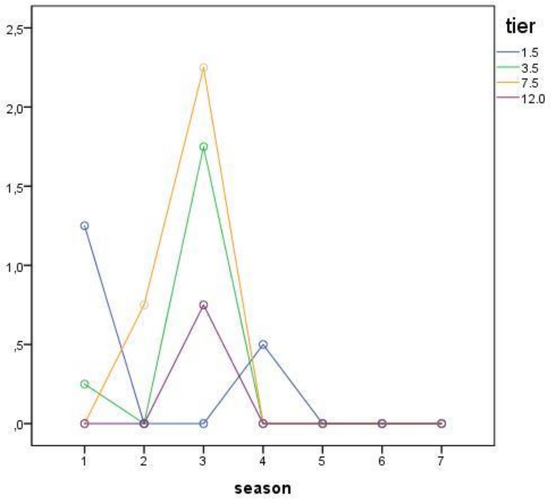

Drosophila histrio

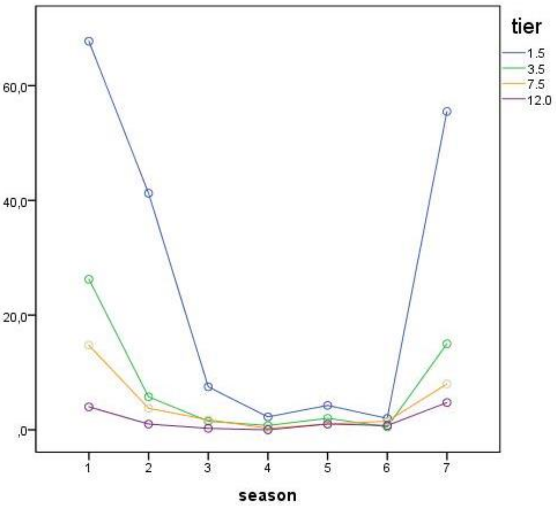

Drosophila kuntzei

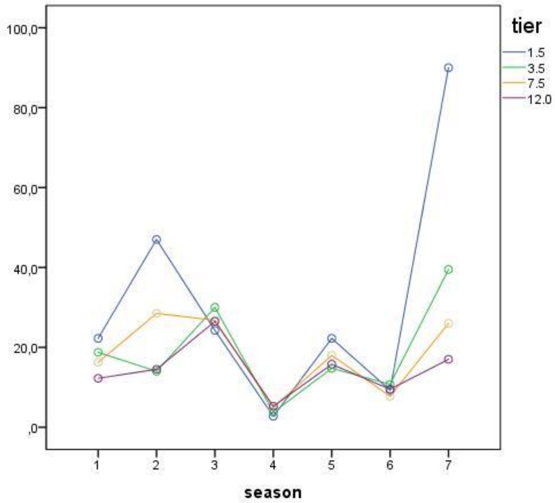

Drosophila melanogaster

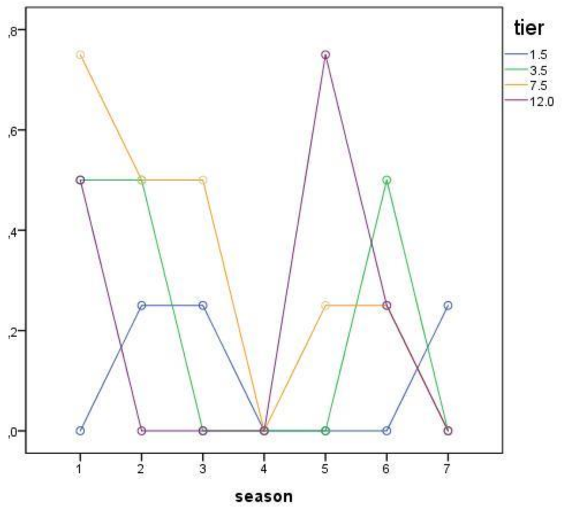

Drosophila obscura

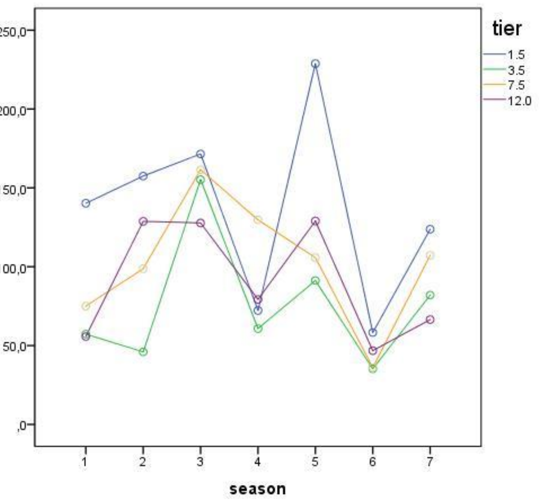

Drosophila phalerata

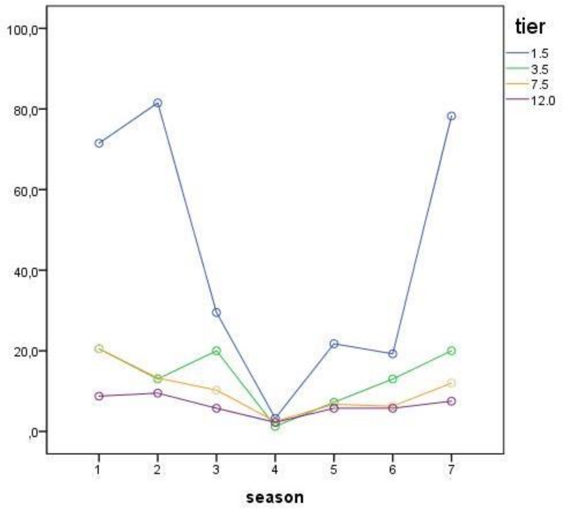

Drosophila subobscura

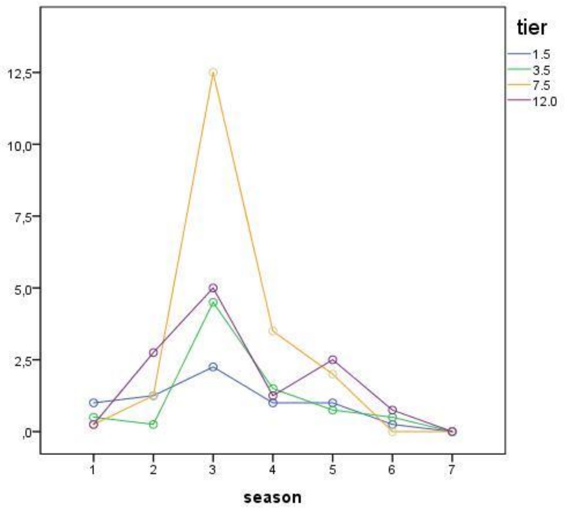

Drosophila subsilvestris

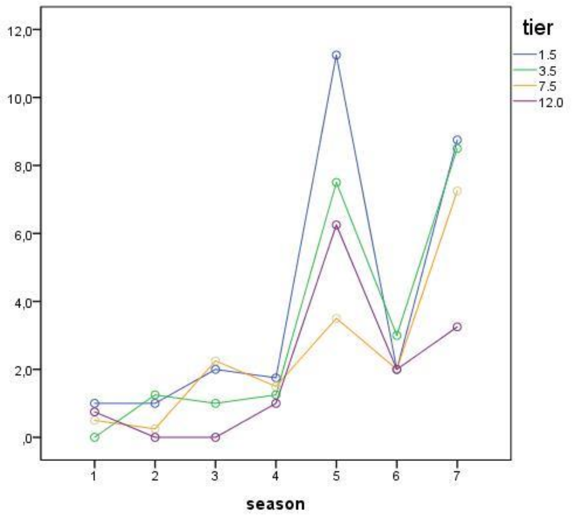

Drosophila testacea

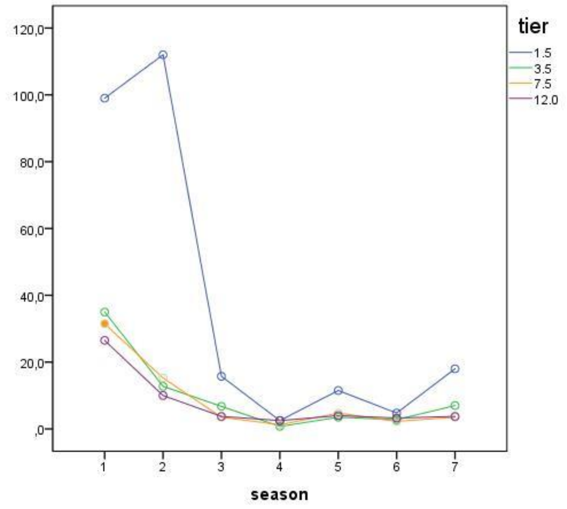

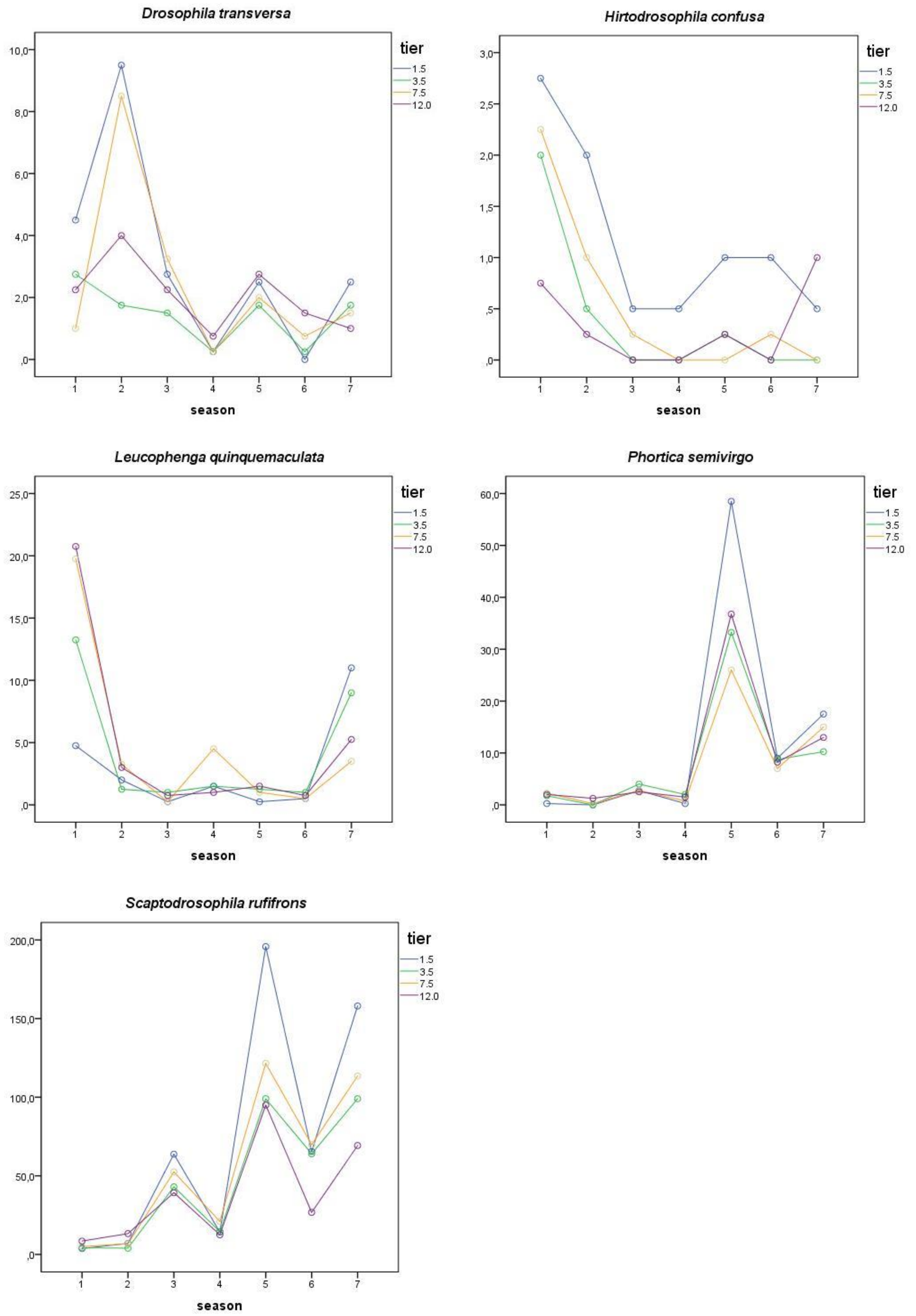

Figure S1. Seasonal changes in the abundance of drosophilids depending on the tier. There are seven collecting periods along the abscissa axis, from the first half of June to mid–September. On the ordinate axis are the marginal average numbers estimated by MANOVA. Lines corresponding to a certain tier are marked with color – 1.5 m – blue, 3.5 m – green, 7.5 m – gray, 12 m – purple. Species collected in an amount of less than 20 individuals are not represented.
